# Primary care nurses’ perceptions and experiences of patients being overweight or obese as well as visions and attitudes about working with lifestyle issues: a qualitative interview study

**DOI:** 10.1186/s12912-021-00685-1

**Published:** 2021-09-15

**Authors:** Marie Bräutigam Ewe, Cathrine Hildingh, Jörgen Månsson, Marie Lydell

**Affiliations:** 1 Department of Public Health and Community Medicine/Primary Health Care, Sahlgrenska Academy, University of Gothenburg, Gothenburg, Sweden; 2 School of Health and Welfare, Halmstad University, Halmstad, Sweden

## Background

In 2016, overweight and obesity worldwide was high and obesity rates are expected to increase further until at least 2030 [1, 2]. In Sweden, 51 % of adults were overweight or obese in 2018 [3]. Overweight and obesity contribute to a large proportion of lifestyle-related diseases and increase the burden on the health care system [4]. The obesity “pandemic” in almost all countries seems to be driven by changes in the food system, which is producing more processed, affordable food than ever before [5].

The urban environment has many physical features that reduce the need for physical activity, such as elevators, escalators and other labour-saving devices, along with passive entertainment such as video games and TV watching [6]. This sedentary behaviour has caused what is called a sitting disease (defined as metabolic syndrome and other ill-effects) and is a greater threat to public health and mortality than smoking [7], according to a large cohort study, maintaining physical activity from adolescence into later adulthood was associated with an up to 36 % lower risk for all-cause mortality [8].

In the Ottawa Charter, health care was emphasized to be of particular importance as an arena for health promotion because of a range of contact with the overall population [9], but it seems to be difficult to reorient health care towards health promotion [10]. However, primary care (PC) is the first point of contact for all patients and is thus viewed as the ideal place to address overweight, obesity and lifestyle issues [11]. There are therapeutic recommendations regarding how to treat overweight and obesity in PC [12, 13], nevertheless, it seems challenging for health care professionals to face obesity; according to one study, they are experiencing this dilemma because the patient is recognized as central in disease management but is also unwilling to change, which is a major potential barrier to treatment [14].

In a Swedish study, 73 % of all health professionals in PC would like to work more to support the promotion of healthy lifestyles among their patients, and nurses were most favourable towards this work [15]. Nurses in a primary health care centre (PHCC) have two key roles in obesity management. First, they work with overweight patients with comorbidities; second, they work with healthy overweight patients [16].

PHCCs has guidelines to follow by the National Board of Health and Welfare for lifestyle counselling focusing on alcohol, smoking, unhealthy eating habits and inadequate physical activity for patients at risk (e.g., overweight, diabetes and high blood pressure) [17]. According to a two-year study of health care professionals about their use of practical guidelines, nurses were the professionals who had increased their lifestyle counseling most, however, there was room for improvement concerning methods for reducing alcohol consumption and unhealthy eating habits [15].

Overweight is a sensitive topic, and patients who are overweight often experience stigmatization and discrimination [18]. It is also known that there could be a weight bias in the interaction between healthcare professionals and the patients [19], therefore, it is important to create a climate with empathy and compassion where the patients feel seen and listened to [20]. A recommended method for lifestyle conversations is motivational interviewing (MI) [21]. The method seemed especially suitable for patients who have self-reported that they are motivated and aware of their role in making lifestyle changes. MI can enable patient self-determination and create a sense of well-being [22].

However, there are obstacles to overcome to address obesity in PC. A review of studies about physician and nurses’ approach to overweight and obese patients showed that health advice is more likely to be given when Body mass index BMI increases and can be of poor quality due to educational barriers and availability of resources [23, 24]. These aspects require ongoing education for nurses and other practitioners about raising the topic with patients in a non-stigmatizing and non-harmful way [25, 26].

Most nurses in PC are receptive to and play a key role in health promotion work and are well suited to work with this patient group. It should be clarified how this work can be better organized for the future from the nurse’s point of view to be able to slow down the development of obesity and metabolic diseases as well as improve public health.

### Aim

To describe primary care nurses’ experiences of patients being overweight or obese, as well as primary care nurses’ perceptions of overweight problems in society and visions working with lifestyle issues.

## Methods

### Study design

The study had a descriptive design with a qualitative method and an inductive approach. Semi-structured face-to-face interviews were conducted with thirteen practice nurses in the southwest of Sweden.

### Participants

The inclusion criteria were nurses in PHCC with experience of working with overweight patients. The exclusion criteria were to understand the language in speech or writing. The participants in the study were nurses in a PHCC (registered nurses, health nurses or diabetic nurses). A strategic selection was used regarding age, work experience, gender and private or public workplace. Twelve of them were women aged 27–61 years, and one was a 62-year-old man; the median age was 51 years. Their working experience varied from 2 to 40 years with a median of 28 years. They were recruited from seven primary health care centres (PHCCs) located in the southwest of Sweden. Some PHCCs had more patients with foreign backgrounds and lower socioeconomic situations. The PHCC’s also differed in size and whether they were located on the countryside or in the city. Each head manager of the PHCC received an e-mail with an information letter about the study, and they were asked if they were interested in participating in the study. They were also asked to forward the e-mail to those nurses who worked with lifestyle issues with a focus on overweight. A reminder was sent out after ten days if no one had responded. Thirty-five PHCCs in southwest of Sweden received an e-mail. When the nurses agreed to participate, we conducted an interview at their workplace. One nurse was interviewed in her home. They received both verbal and written information about the study and a consent form to read and sign when we met before the interview.

### Data collection

Data were collected using an open qualitative interview [27] in the form of a dialogue.

The recruitment occurred between October 2019 and February 2020. A semi-structured interview guide developed by the authors was used with open questions, and the interviewer also had the opportunity to ask probing questions. The first interview was carried out as a pilot, and then included in the study. The topics were nurses’ thoughts about overweight in general and in society, why overweight has increased, what experiences they had working with patients with overweight and lifestyle difficulties, how they worked with lifestyle issues and if overweight was prioritized in PHCCs. Furthermore, there were questions regarding if they received support from the manager, if there were ethical dilemmas, what results they obtained and if they had any dream scenarios of how they would like to work with obesity and lifestyle issues. The interviews lasted from 22 to 45 min and were digitally recorded and transcribed verbatim. Notes was also taken during the interviews. Data saturation was achieved when no new data occurred in the interviews.

### Data analysis

Qualitative content analysis was conducted based on the steps described by Graneheim & Lundman [28]. The analysis began by reading the transcriptions several times to obtain an overall sense of the data. Sentences and phrases corresponding to the aim of the study, referred to as meaning units, were highlighted. The meaning units were abstracted, coded and subsequently sorted into subcategories and categories based on similar content of the analysis, an example can be seen from table 1 (below). The subcategories and categories were discussed in a cross-professional group with four people (two nurses, one physiotherapist and one physician). Throughout data analysis, several meetings of this cross-professional group occurred. All members in the group had read the interviews and discussed the codes, subcategories and categories until a consensus was met. They all had long experience of working with lifestyle issues and of the analysis method. The translation into the English language occurred after the data analysis. The analysis of the interviews resulted in three categories and nine subcategories.

Examples of the analytic process in the categories, Primary care nurses wish to promote health and prevent illness, Arenas for health promotion in society and support patients to change their behaviour, follows in Table 1.

**Table 1:** Example of the analytic process for each of three categories

Meaning unit	Condensed meaning unit	Code	Subcategory	Category
It is important to talk in a good way so that you do not offend the person in front of you and encourage what is good; do not to complain too much about what does not work… do not use pointers because it is very sensitive to discuss overweight due to the psychological, social and other implications.	It is important to talk so that you do not offend the patient and encourage what is good and do not use pointers. Overweight is loaded mentally and socially.	encouraging patient meetings	A good patient meeting creates conditions for lifestyle changes.	Primary care nurses wish to promote health and prevent illness.
One might think that the food market, the stores have a great responsibility that takes in all these unhealthy goods and how they are then distributing them in the store. If you go shopping and are hungry, it is difficult to resist and you might just grab a bag of sweets on the way out.	The food market has a great responsibility that takes in and distributes unhealthy goods in the store. If you shop hungry, then maybe you grab a bag of sweets.	The responsibility of the food market	Regulation of the food market	Arenas for health promotion in society
You feel if you have worked for a year with a patient and no change happens. So, then you have to pause and give them some time to mature. They have to somehow land in their own motivation. They need to build a greater self-motivation and a greater confidence in their own ability to cope with making a change in their lifestyle.	If you have worked with a patient for a long time and nothing happens, you have to pause and let the patient find their motivation and confidence in their own ability to make a lifestyle change.	To sometimes paus the patient visits	The patient’s motivation determines the outcome	Support patients to change their behaviour

## Results

The analysis of the interviews resulted in three categories and nine subcategories which is presented in Table 2.

**Table. 2:** Overview of Subcategories and Categories

Subcategories	Categories	Code	Category
Schools and media's impact on lifestyle	Arenas for health promotion in society		
Regulation of the food market			
Health promotion outside of the health care mission			
A good patient meeting creates the conditions for lifestyle change	Primary care nurses wish to promote health and prevent illness		
Lack of knowledge how to implement guidelines			
Ethical and cultural issues challenge the nurse's role			
Tailored interventions as a meeting point for patients			
The patient’s motivation determines the outcome	Support patients to change their behaviour*.*		
The individual has to choose a healthy lifestyle			

### Arenas for health promotion in society

This category consisted of three subcategories: *School’s and media’s impact on lifestyle*, *Regulation of the food market and Health promotion outside of the health care mission*

#### Schools and media’s impact on lifestyle

The nurses found it important to inform parents, children and teenagers early about what is a healthy lifestyle. School was considered an important platform, and the school nurse had a task to begin early when informing children and young people of what a healthy diet is in order to establish a normal eating behaviour. Schools should teach students to think critically about what to listen to and how to screen information from Instagram and Facebook. The nurses experienced that adolescents were adversely affected by images of a perfect body that led to eating disorders such as bulimia and anorexia. There was also experiences that the school should work more on encouraging more daily exercise and preferably encouraging gymnastics between lessons so that the students could develop a healthier lifestyle, prevent overweight and perform better in school. Information on healthy diets and exercise was considered important to disseminate. A nurse said the following:

*“School is a place for information and thoughts on what is normal diet and exercise…. a little more wellness at school does not hurt.”* (Nurse 11).

The nurses felt that the media such as TV, magazines and social media had an increasing impact on our lifestyle and our eating habits and that different messages in newspapers and magazines about what is a healthy diet and lifestyle became confusing. The influence of the media on our lifestyle, especially our eating habits, was something that was reflected around, and many felt that there was far too large a selection of different food programmes on TV with “delicious” food and far too few educational and informative programmes about what is a healthy diet and how to cook it. According to a nurse,

“*All these food programmes on TV. It is senseless, everything is fixated on food. Television should show programmes about what a normal portion looks like instead of yummy food.”* (Nurse 3).

It was also perceived that advertising for sweets and fast food was occurring far too often and had a negative impact affecting everyone to crave unhealthy food and eat too much especially if they were already feeling bad and were more easily affected.

#### Regulation of the food market

Some of the nurses felt that actors such as the food industry and food stores made it difficult for individuals to know what a good and bad diet is. It is often the responsibility of the individual to resist sweets at the checkout. The nurses were told by several patients that it was a challenge to shop when they were hungry and stressed, and it was then difficult to resist unhealthy food. The availability of fast food and the fact that the stores were open around the clock were considered problems that made it difficult for individuals to make the right choices. A nurse pointed out the following:

*“It is very easy to choose wrong because the food industry produces food with too much fat and sugar, and it becomes difficult to know what a healthy diet is without spending a lot of time on it.”* (Nurse 1).

The nurses described that the grocery stores should take greater responsibility and not put candy at the checkout because it became a major challenge for individuals to be attracted to wrong choices. Two groups that were particularly vulnerable were people with a foreign background and those with poor finances who often bought two large bottles of Coca Cola instead of one because it was marketed as being cheaper. The nurses expressed that society’s government should do more to strengthen public health in school and promote outdoor environments and reduced consumption of sugar in the community to protect the most vulnerable groups. They described that society should raise the issue of introducing a sugar tax and lowering the price of healthy food. A nurse said the following:

“*A sugar tax would have been good, but then you should lower the price of healthy foods like fruits and vegetables.”* (Nurse 9).

#### Health promotion outside of the health care mission

The nurses agreed that private actors (outside PHCC) were needed to improve and maintain public health. For children and young people Generation Pep ® was mentioned as those who work for children and young people’s health who are also active in the school. The most common and popular actors for diet were Weight Watchers ® (WW). Some nurses encouraged patients to help with the diet with the help of a WW app where they could receive coaching and support every week that the nurses did not always have the opportunity to offer. The nurses’ experienced that all patients were at different levels, and some were already using WW and it was enough to follow up with them every six months. For others, they wanted the nurse’s support and to come and weigh at the PHCC. Some of the nurses described that it was important not only to talk but also to actively invite patients to training. One of the nurses had started a training group for the patients outside the PHCC at a training centre, and several patients accompanied her. A nurse said the following:

“I believe in not only talking but also showing. - You, on Wednesday we will see you *at the gym in the entrance. Then, you can have a free lesson. Over the years I have supported some 30 patients to develop a new lifestyle with exercise.”* (Nurse 13).

At a PHCC, a collaboration with WW had been tried by the health centre by recruiting a number of patients who wanted to be part of a group, but the collaboration dissolved because there was some discussion about whether it was right or wrong. A nurse described it as follows:

*“It was not that good. A public health centre that recruits patients to a private operator; what about all the other operators available then? I know there were discussions about this.”* (Nurse 7).

In some areas with a poorer socioeconomic status, the nurses felt that a barrier for patients going to the gym. The nurses felt that it would be good for some people to come out and meet others by training together, but the nurses described that some people managed to work out on their own with the help of various gym passes on TV or via the computer.

### Primary care nurses wish to promote health and prevent illness

This category has four subcategories: *A good patient meeting creates conditions for lifestyle changes, Lack of knowledge about how to use guidelines, ethical and cultural problems challenge the nurse’s role and Tailored interventions as a meeting point for patients.*

#### A good patient meeting creates the conditions for lifestyle changes

A good meeting with the patient was important for the nurses in building a relationship. It was perceived as a prerequisite for the patient to succeed with weight loss and a change in lifestyle. The nurses described that the care had changed and in ten years, the client contact had decreased, and the administrative work increased. The nurses experienced that prevention work not was prioritized and that the money was saved here and now, but in the future, the nurses believed that it would be more expensive for PHCCs if the preventive and promotive work is not given more priority. According to a nurse,

*“We must be allowed to have more nurse visits. We need to stop the development in the early stages. We cannot just cure the sick patients because we have to work more on this as well.”* (Nurse 12).

Most nurses would have liked more time for this work because there was a lot to do, and the time available was sometimes not enough. During the first visit there could be things that affected the patient’s life situations such as divorce, work situations and childhood events. These experiences affect behaviour, and sometimes the nurses felt similar to conversational therapists. A nurse described it as follows:

*“The first visits need to be 60 minutes to go through everything; many may have a lifestyle that affects them. You are like a conversational therapist.”* (Nurse 2).

The nurses said that the manner of talking with the patients played an important role in bringing about a change. Since it was a delicate subject and they could feel violated, it was important not to use reprimands. The nurses described that they used a variety of motivational conversations with open questions but did not always use the available technology to the full extent, and some wanted to have more education. Some nurses described that a challenge in the work was that at some PHCCs, the patients were sometimes very well prepared and educated. They had read on the internet and thought they knew what was good, and there was much discussion about different diets necessitating that the nurse balances the conversation. A nurse pointed out the following:

*“There are many challenges. Here, many are well educated and do not want to listen to a nurse who tells them what to do and what not to do.”* (Nurse 7).

The nurses felt that adapting the efforts to each individual’s life situation and conditions and gently taking care were important to achieve a sustainable result. A nurse said the following:

*“When I am stressed and go too fast with dietary advice, then resistance comes from the client, and I have to back track a little. The mood changes, and it’s a dance all the time.”* (Nurse 8).

#### Lack of knowledge how to implement guidelines

Several nurses described that they educated themselves to become district nurses in order to do more preventive or promotive work, but it has not become as much as they wanted. The nurses described that they wanted to inform individuals as early as possible (child care centre), and parent groups were considered; maternal care was a good opportunity to decrease the development of overweight in society. They felt that there were guidelines for different conversational efforts during lifestyle conversations and for overweight, but it was unclear how to set it up; they felt that everyone worked differently on this. The nurses had the experience that at several PHCCs, there were no dietitians available, and they sometimes felt that for some more difficult patients they needed slightly more education about diet. A nurse pointed out the following:

*“Having a dietician in this work would have been worth gold.”* (Nurse 12).

They expressed their desire for training in conversational methodology and psychology adapted to different personality types of patients in order to personalize and obtain better results. They felt that they had to read on their own and call colleagues around and ask each other. A nurse described it as follows:

*“I would love to go on a diet education. So, you absorb everything and try to read a lot of yourself. You would have liked to go.”* (Nurse 2).

Another nurse described it as follows:

*“What I miss very much is that there are no guidelines for health conversations in local health care, so we invent the wheel at all health centres. How to set it up, how many times, at what rate, etc. Everyone works differently.”* (Nurse 5).

The patient materials used in the work were often the region’s own materials, plate models and the Swedish Food Agency’s advice.

#### Ethical and cultural issues challenge the nurse’s role

The nurses described ethical difficulties and challenges that they had to deal with in their work with overweight patients. One nurse had preferably recommended another diet that she herself believed in and felt it was difficult for her not to talk about it. Other nurses experienced that there were many different diets and sources of dietary advice in circulation, but everyone agreed that it was important that the PHCC work be evidence based. The nurses described that PHCCs should be able to do more for those who needed it. The nurses felt that it could be a culture crash with foreign-born groups, especially for women from a culture where it was considered nice to be overweight and that it was associated with wealth. It was challenging for the nurses to manage them. Some described that it was unethical not to inform the patient because they needed to gain knowledge that being overweight is a health risk. A nurse said the following:

*“I do not think you should be afraid to tell the patient that in order to get well in the knee you must lose weight before the surgery. Otherwise it is a waste of money and resources.”* (Nurse 11).

The nurses felt that it was easier to address the overweight problem if the patient already had high blood pressure because then you could go around the whole thing and start focusing on other factors such as sleep and stress to get into diet and overweight at a later stage. A nurse was worried that the patient would find it difficult that she was a normal weight and had no experience of being overweight; thus, she was careful to proceed cautiously. A nurse described it as follows:

*“It is important to talk so that you do not offend the patient and encourage what is good and do not use reprimands. Overweight is loaded mentally and socially.”* (Nurse 7).

Some nurses talked about the experience of meeting patients who should lose weight before an obesity surgery and who they met far later when they had lost weight but life had not become as they imagined. It was difficult to know how to support them. A nurse described what a patient told her regarding his thoughts about the situation:

*“You have operated on my stomach, but you have done nothing to my mind and my brain.”* (Nurse 1).

#### Tailored interventions as a meeting point for patients

One nurse described that their PHCC had profiled itself as a healthy PHCC and wrote on its website that health is not something you can take for granted, but it takes a great deal of work to achieve good health. The nurses had varying wishes and visions on how to set up work with overweight and lifestyle to improve compliance and obtain better results. They felt that they had a good working relationship with the physiotherapists already, but most lacked a broader and more structured team collaboration with more professionals such as a psychologist, especially for patients with mental health problems comforting themselves with sweets, getting a sugar kick, they were experienced as difficult patients. The nurses expressed that the patients themselves could choose whether they wanted individual visits or group interventions. Preferably there would be a greater selection of efforts than previously to choose from. The nurses described that in most places, there were no overweight groups at present, but this was something that several nurses wanted to start. A nurse described it as follows:

*“Teamwork among staff and groups is necessary if you have a dream. Then, patients can advise each other and motivate each other.”* (Nurse 12).

There was a desire for more time, support and resources for this work to be able to check on follow-up visits to a greater extent. The nurses wanted to provide effort earlier, especially for individuals with pre-diabetes to prevent the development of diabetes. A lifestyle reception with lectures as a meeting point for patients with a “preventive approach” that was more structured and focused on this were proposed. A nurse described it as follows:

*“I would like a lifestyle reception where you could take them on closer visits, have motivational conversations and follow up more often. There is lots of money to save, as well as suffering, and we can help patients be happy.”* (Nurse 1).

The nurses described wanting to have availability during the evenings for a health school with tailor-made material with lectures for those who had difficulty coming during the day. A nurse described that her PHCC intends to introduce video meetings to reach the patients and perhaps also to increase the availability of lifestyle calls. Another nurse would like to set up these services in her own way and start a cooking school where the patients can learn how to cook a healthy diet. Health examinations were described by some nurses as belonging to PHCCs and were thought to be a good way to prevent illness but were mostly offered at private PHCCs. The nurses thought that PHCCs should try to reach out to the population more with health information before overweight/obesity went too far. The nurses described that they also lacked a forum to meet and exchange similar experiences among diabetes nurses. They preferred to have lecturers, continuing education and information on the latest knowledge about obesity and lifestyle from reliable sources such as studies and reports from all over Sweden. A nurse pointed out the following:

*“We have known that we need to know more about this. You want to feel safe in your profession so that you can talk to this patient about this; it needs to comes from safe sources, and you want not only the VG region but also nationally in Sweden to have more strength in what we say.”* (Nurse 11).

At some PHCCs, they worked more actively with prescriptions for physical activity on prescription (PaP) to the patients to get them started with training, and in some places, there was the possibility of referral to clinics that specialized in supporting people with physical activity at a low cost to the patient. Most nurses felt that it was a good tool that could be used more. At some clinics, it was more common for physiotherapists to prescribe PaP.

### Support patients to change their behaviour

This category has two subcategories: *The patient’s motivation determines the outcome and the individual has to choose a healthy lifestyle.*

#### The patient’s motivation determines the outcome

The motivation of the patients varied depending on their life situations and played a major role in the results obtained. The nurses described that several patients lacked motivation and had not arrived voluntarily but at the doctor’s advice. Some were more motivated when they had high blood pressure or diabetes, and others were not. The nurses found it difficult to know when to give up and when to continue the conversations. Motivating patients to change their lifestyles could be challenging, tiring, comfortless, heavy and difficult. As a nurse, you often experienced resistance from the patient to change and because the patient’s motivation goes up and down; sometimes no one came anywhere, and it was not easy to know when to pause the visits. A nurse mentioned:

*“It is difficult to penetrate all defence mechanisms when they are not motivated and yet give them time. It is a balance: when to give up and when to move on.”* (Nurse 5).

The nurses also experienced the work alternatively as fun, rewarding and enriching, especially when the patients had good results and felt better. Some patients had several poor living habits, and if they could get rid of at least one of them, they often felt much better, and then their motivation increased. According to a nurse,

*“One patient thanked me for beginning to understand that she needed to relax a bit and eat slowly and not eat while walking.”* (Nurse 3).

Another nurse said,

*“This can be difficult to motivate. However, it is wonderful when you manage to get a person on the train, and they have succeeded in losing weight and started to move more. I can be a mainstay, but I can never be part of everyday life.”* (Nurse 9).

#### The individual has to choose a healthy lifestyle

The nurses described that as individuals, the patients had their own health responsibility, but that they have different presumptions depending on their education, economic situation, previous illnesses and mental health. They described that we have become more sedentary in front of computers and that this begins early in childhood. The nurses felt that parents should take greater responsibility and not sit with their smart phones themselves. A nurse described it as follows:

*“The parents have a responsibility in raising children; they drive the children everywhere, but they have to make sure that the children start to move more.”* (Nurse 5).

The nurses described that as a parent, one must be a good role model in terms of diet, exercise and social media and live as one learns. A nurse said,

*“It is important to introduce your children and grandchildren early in training so that they develop habits for life.”* (Nurse 13).

One problem that the nurses perceived was that there was much information available, and it could be difficult to screen for what is good or bad; they felt that PHCC has a responsibility to work with evidence-based information and convey what is good.

The nurses felt that the individual’s choices were influenced by knowledge about good food, lack of time and stress such as being in a hurry when shopping and not being able to read on the packaging. Socioeconomics and education also played major roles, and in some cases, foreign background leading to a lack of knowledge played a role; it was not uncommon to mix sugar in the milk for babies. Two nurses said,

*“Cheap food is unhealthy, and patients lack knowledge on how to cook cheap and healthy food and think that everything should go fast.”* (Nurse 3).

*“I try to get people to stop with fast food and buy ordinary food like meat, potatoes and vegetables and cook themselves. For those who say they ate dinner at McDonald’s, I say it is no use because it is just “junk” you have eaten.”* (Nurse 13).

The nurses perceived that the individual wanted to blame other things. They felt that the patients had their own responsibility for their choices, but the nurses felt that they had a mission to strengthen the patients to take responsibility for their lifestyles both in thought and action. They also expressed that the individual had a responsibility to seek help when the problems became too large and affected the quality of life. Two nurses described it as follows:

*“The individual has the main responsibility to find out the facts about what is good and bad and react when he or she cannot do the things he or she wants because of being overweight. The individual has to react when he or she is cut off in his or her life and seek help.”* (Nurse 13).

*“An important task is to get patients to focus on their lifestyle and not just their medication to feel good.”* (Nurse 12).

The nurses described that when patients felt that they were feeling better about their lifestyle change, it was easier for them to keep up and harder to go back to their old lives.

## Discussion

The nurses identified that weight management and lifestyle interventions are an important part of their role and they believed that PHCC is an ideal place to work with this problem, but it is complicated by high workload, educational deficiencies, and societal factors. The nurses had visions of a multidisciplinary team working with this patient group in PHCC and for more individualized care and efforts with group meetings, lectures and digital solutions.

To make the environment less obesogenic, nurses recommended increasing the prices of unhealthy food and beverages. Like the World Health Organization (WHO), they identified a number of actions that the food industry could take to improve the population’s nutrition by limiting the levels of fat, sugar and salt in products and practising responsible marketing [29]. Another subject the nurses brought up was the marketing of unhealthy food in all sorts of media, they thought that children and parents should be encouraged to develop their critical thinking about television food advertising [30]. However, the school nurses have an important role and can be more involved in education of pupils about these issues.

There is a gap between the nurses’ desire to work more with health promotion and prevention and the available resources, which also another study pointed out [25]. As in our study, it has been shown previously that nurses considered MI as a good tool that is helpful and non-judgemental and puts nurses and patients on the same level. However, sometimes because of a lack of knowledge, training, and education, it was not fully used [31], but with continued MI-education offered in the regions, and the right conditions at the workplace, more nurses could benefit from MI.

Several nurses had limited education on the guidelines and how to use and implement them, and they believed that methods of working varied between different PHCCs. Although one study showed a certain improvement after two years regarding the use of guidelines, more improvements seem to be needed [15]. In our study, one way to discuss the problem from the nurses’ point of view was to start a forum for those nurses who had worked with overweight and lifestyle issues nationally in Sweden. Bringing up the question about to start a lifestyle forum for nurses nationally could be handled at the collegiate meetings to start with.

The nurses also described some ethical dilemmas they encountered in their work with overweight individuals regarding their own weight status and attitudes. Another study also showed that nurses own weight status could be a potential barrier to raising the issue [26]. It is important for nurses to have this in mind, be aware of the problem and not let it stop them from discuss the topic. As in previous research [32], the nurses in our study experienced an increased number of internet-read patients, and this was a new challenge for them to handle these patients. As social media gain a greater influence, it is important that nurses learn to critically review facts in their education, so that they can respond to untrue statements. It is also valuable that newly educated nurses can share their knowledge with nurses who have worked for more years in the profession. Other issues occurred especially in women with foreign backgrounds and low socioeconomic status (SES) who fed their children and themselves unhealthy food and sweets [33]. It was difficult for the nurses to motivate them to lose weight, and it was a challenge not to use reprimands. If PHCCs could develop more groups for overweight individuals, it may be an idea to suggest that these patients participate and receive inspiration from others.

According to previous research [34], a health-promoting perspective leads to a shift from costs to human concerns, as well as a shift from focusing on problems and risk factors to seeing possibilities, resources and factors that keep people healthy. Even so, the focus in primary care is still more disease- oriented. Anyway, nurses have an important role in health promotion work, especially considering the knowledge they possess regarding lifestyle issues. Questions how to make nurses knowledge more visible and how their knowledge can benefit the public could be addressed.

### Strengths and limitations of the study

To strengthen the credibility, the strategic selection provided a good geographical and socioeconomical distribution of the PHCCs, and the nurses were of different ages and all had different working experiences. They also had slightly different professions, and they had experience in working with overweight individuals. There was only one male nurse, but this reflects the reality in PHC. According to the transferability, the context is in PHCCs with several nurses working there. We used a strategic selection, and it was difficult to recruit participants for interviews. This reflects the situation for nurses in PC who have a pressed schedule and perhaps a lack of interest in research. This may have resulted in nurses who were most interested in overweight and lifestyle issues being chosen to participate, and we did not reach those with another opinion; this could be a weakness. We had several open semi-structured questions to capture everything we wanted to identify and included probing questions. The fact that the shortest interview was not more than 22 min was due to the nurses’ time limits in PHCCs. The nurses had tight schedules, and all the interviews were performed at their workplaces, with one exception. According to transferability, we have chosen to present a table to follow the interpretation of the text with meaning units, condensed unit, codes, sub-categories, and categories. The findings are illustrated by representative quotations in the text to help the reader to understand and assess the trustworthiness of the analysis. Nevertheless, we felt that the material was rich in content and answered the aim of the study.

To achieve trustworthiness, such as credibility and dependability, the analysis was performed with help of all authors by reading the interviews and meeting several times to discuss further and reach a consensus. As in most qualitative studies, the researchers bring prior understanding because of their knowledge and previous experience with the subject. This can lead to both disadvantages and advantages when interpreting the results. Despite this, the researchers tried to stay as objective as possible in the interpretation. The data were collected for a fairly short period of time; therefore, there is no risk that it will change, and that strengthens the dependability.

## Conclusions

According to the nurses, too little has been done to slow down the development of overweight and obesity by society and at a governmental level. Food industry and food stores make it difficult for individuals to choose healthy food and therefore a regulation of the food market is of utmost importance. Primary care plays an important role to reach out with health information. As nurses meet parents in child care centres, children and young people in school, as well as patients of all ages in primary care, they have a great opportunity to build trusting relationships and use motivational conversations and thereby create conditions for lifestyle change. However, there is a need for team collaboration in primary care where different professionals take part in promotion health and preventing disease. Continued research in the area could involve interviewing decision makers or managers in the regions about their strategies to give promotion work a higher priority in Primary care.

## Supplementary Information

**Additional file 1:** Interview Guide Description of data: List of questions for the participants in the study.

**Additional file 2: **COREQ 32-item checklist

## Data Availability

The dataset generated during the study will not be shared due to participants’ anonymity and confidentiality and to respect for the participants’ sensitive contribution. The data are with the corresponding author but are available from the corresponding author on reasonable request.

## Abbreviations

MI: Motivational Interviewing
PC: Primary Care
PHCC: Primary health care centre
PHCCs: Primary Health Care Centres
