# Supplementary material for: Primary care nurses’ perceptions and experiences of patients being overweight or obese as well as visions and attitudes about working with lifestyle issues: a qualitative interview study
Source: BMC Nurs. 2021 Sep 15;20:170. doi: 10.1186/s12912-021-00685-1 (PMC8442465; doi:10.1186/s12912-021-00685-1)
Supplement: Supplementary file 1 — Additional file 1: Interview Guide Description of data: List of questions for the participants in the study. [file 12912_2021_685_MOESM1_ESM.docx]

**Interview Guide**

**Opening question**

Tell us about yourself and what you think are your most important tasks at your PHCC?

**Introduction question**

How do you think about over weight in our society?

Who do you think is responsible for this type of issues?

**Questions**

How do you work with lifestyle issues with a focus on overweight at your health center?

What difficulties and ethical dilemmas do you experience in this work?

What are your previous experiences in working with overweight problems?

If you were to wish, how would you like to work with lifestyle issues in overweight people?

How do you feel that these issues are prioritised in your work at your health center?

What support is there for you working with these issues in your workplace?

If you were to wish, which other actors do you see could work on these issues?

How do you experience working with lifestyle issues in overweight people?

How do you feel about the competence level with the staff who work with lifestyle issues with a focus on overweight?

What results do you feel you get in your work with lifestyle issues with a focus on over weight?

How do you reach the patients who need help to change their lifestyle with a focus on over weight?

**Final question**

Is there anything you would like to add?
