# Supplementary material for: Primary care nurses’ perceptions and experiences of patients being overweight or obese as well as visions and attitudes about working with lifestyle issues: a qualitative interview study
Source: BMC Nurs. 2021 Sep 15;20:170. doi: 10.1186/s12912-021-00685-1 (PMC8442465; doi:10.1186/s12912-021-00685-1)
Supplement: Supplementary file 2 — Additional file 2: COREQ 32-item checklist [file 12912_2021_685_MOESM2_ESM.docx]

COREQ 32-item checklist

No Item Guide questions*/*description

Domain 1: Research team and reﬂexivity

Personal Characteristics

1. Interviewer*/*facilitator Which author*/*s conducted the interview or focus group?

The research nurse/Ph.D. Student.

1. Credentials What were the researcher’s credentials? *E.g. PhD, MD*

Professor, MD/Professor, PhD.

1. Occupation What was their occupation at the time of the study?

Professor, Hospital CEO, Senior Lecturer.

1. Gender Was the researcher male or female?

Female.

1. Experience and training What experience or training did the researcher have? Good experiences in data collection, analysis and in the area of lifestyle interventions.

Relationship with participants

1. Relationship established Was a relationship established prior to study commencement?

No previous relation.

1. Participant knowledge of the interviewer

What did the participants know about the researcher? e*.g. personal goals, reasons for doing the research*

Information letter along with the invitation to participate.

1. Interviewer characteristics What characteristics were reported about the interviewer*/*facilitator? e.g. *Bias, assumptions, reasons and interests in the research topic*

Nothing else but nurse and PhD student.

Domain 2: study design

Theoretical framework

1. Methodological orientation and Theory

Participant selection

What methodological orientation was stated to underpin the study? *e.g. grounded theory, discourse analysis, ethnography, phenomenology, content analysis*

Content analysis, to be found under Data analysis line 138.

1. Sampling How were participants selected? *e.g. purposive, convenience, consecutive, snowball*

Strategic selection, to be found under participants line 103.

1. Method of approach How were participants approached? e*.g. face-to-face, telephone, mail, email*

Face to face, to be found under Study design line 98, Participants line 118 and under Data collection line 123.

1. Sample size How many participants were in the study?

13 participants, to be found under Study design line 98.

1. Non-participation How many people refused to participate or dropped out? Reasons? 35 PHCC were approached with e-mail, 19 did not answer, 3 declined and 10 agreed to participate. No drop outs from those who agreed to participate. To be found under Participants line 113-117.

Setting

1. Setting of data collection Where was the data collected? e*.g. home, clinic, workplace*

At their workplaces and at home, to be found under Participants line 118.

1. Presence of non-participants Was anyone else present besides the participants and researchers?

No.

1. Description of sample What are the important characteristics of the sample? e.g. demographic data, date

A strategic selection was used regarding age, occupation, work experience, gender and private or public workplace, to be found under participants line 103.

Data collection

1. Interview guide Were questions, prompts, guides provided by the authors? Was it pilot tested?

Yes, to be found under Data collection line 124. Yes, first interview regarded as pilot, to be found under Data collection line 126. The interview guide/questionnaire was developed by the authors specifically for this study.

1. Repeat interviews Were repeat interviews carried out? If yes, how many?

No.

1. Audio*/*visual recording Did the research use audio or visual recording to collect the data?

Yes, audio recording to be found under Data collection line133.

1. Field notes Were ﬁeld notes made during and*/*or after the interview or focus group?

Yes, to be found under Data collection line134.

1. Duration What was the duration of the interviews or focus group?

4 months, to be found under Data collection line124. Interviews themselves were between 22 to 45 minutes long, to be found under Data collection line 133.

1. Data saturation Was data saturation discussed?

Yes, to be found under Data collection line 134.

1. Transcripts returned Were transcripts returned to participants for comment and*/*or correction?

No.

Domain 3: analysis and ﬁndings

Data analysis

1. Number of data coders How many data coders coded the data?

2.

1. Description of the coding tree Did authors provide a description of the coding tree?

Yes.

1. Derivation of themes Were themes identiﬁed in advance or derived from the data?

The themes were derived from the data.

1. Software What software, if applicable, was used to manage the data?

MS Excel was used to manage the data.

1. Participant checking Did participants provide feedback on the ﬁndings? No.

Reporting

1. Quotations presented Were participant quotations presented to illustrate the themes */* ﬁndings? Was each

quotation identiﬁed? e*.g. participant number*

Yes, first to be found under Results line 191.

1. Data and ﬁndings consistent Was there consistency between the data presented and the ﬁndings?

Yes.

1. Clarity of major themes Were major themes clearly presented in the ﬁndings?

Yes.

1. Clarity of minor themes Is there a description of diverse cases or discussion of minor themes?

Yes, to be found in the sub categories.
